# Development of a Dog Health Score Using an Artificial Intelligence Disease Prediction Algorithm Based on Multifaceted Data

**DOI:** 10.3390/ani14020256

**Published:** 2024-01-13

**Authors:** Seon-Chil Kim, Sanghyun Kim

**Affiliations:** 1 Department of Biomedical Engineering, Keimyung University, 1095 Dalgubeol-daero, Daegu 42601, Republic of Korea; 2 DDcares Inc., #710 815 Daewangpangyo-ro, Sujeong-gu, Seongnam-si 30119, Republic of Korea; shkim@ddcares.com

**Keywords:** companion dog, acceleration sensor, health, gyro sensor, Health Score

## Abstract

**Simple Summary:**

Typically, individuals who own dogs may not possess veterinary expertise, complicating their ability to promptly discern the health status of their pets. Consequently, these owners often fail to seek timely medical intervention, resulting in the necessity of visiting animal hospitals. To address this issue, our study investigated methods for dog owners to easily and promptly ascertain their dogs’ health status. We equipped dogs with sensor-fitted leashes and monitored their behavioral patterns over a nine-month period. The health status determined through behavioral pattern analysis aligned with veterinarian diagnoses at a rate of 87.5%. We anticipate that future advancements in sensor technology and behavioral pattern analysis will significantly aid dog owners, particularly those without veterinary training.

**Abstract:**

Detecting aberrant behaviors in dogs or observing emotional interactions between a dog and its owner may serve as indicators of potential canine diseases. However, dog owners typically struggle to assess or predict the health status of their pets independently. Consequently, there is a demand for a methodology enabling owners to evaluate their dogs’ health based on everyday behavioral data. To address this need, we gathered individual canine data, including three months of standard daily activities (such as scratching, licking, swallowing, and sleeping), to train an AI model. This model identifies abnormal behaviors and quantifies each behavior as a numerical score, termed the “Health Score”. This score is categorized into ten levels, where a higher score indicates a healthier state. Scores below 5 warrant medical consultation, while those above 5 are deemed healthy. We validated the baseline value of the Health Score against veterinarian diagnoses, achieving an 87.5% concordance rate. This validation confirms the reliability of the Health Score, which assesses canine health through daily activity monitoring, and is expected to significantly benefit dog owners who face challenges in determining the health status of their pets.

## 1. Introduction

The prevalence of households with dogs and other domestic pets has risen in recent years [1]. These animals, known as companion animals, coexist with humans, offering emotional support and forging deep bonds. This trend has led to a growing interest in the health of companion dogs, in parallel with the increasing number of such animals in homes [2]. In human healthcare, the advent of various technologies allows individuals to discuss and monitor their health status using real-time monitoring and AI diagnostic systems, enhancing their quality of life through information and communication technology (ICT) advancements [3]. However, companion dogs primarily communicate through gestures and behaviors, which present challenges for quantitative health status assessment [4]. Dogs predominantly rely on behavior-based communication, necessitating the differentiation between routine, communicative, or disease-induced distorted behaviors. Owners can gauge a dog’s health by noting repetitive and specific behaviors. For instance, a dog’s land smelling behavior can indicate playfulness, stress, or illness. Rapid weight loss may suggest diabetes or kidney disease, while weight gain could indicate overeating or a lack of exercise. Frequent scratching of the eye area might be symptomatic of keratitis or conjunctivitis. However, owners lacking veterinary knowledge may struggle to recognize these specific behavioral or physical changes [5]. Skin diseases, which are common in companion dogs, often recur and require extended treatment, even for simple forms [6]. Notably, life-threatening emergencies can arise from untreated inflammatory skin diseases [7]. Therefore, identifying specific abnormal behaviors is crucial for assessing a dog’s health and providing appropriate treatment [8].

Numerous studies have investigated the prediction of canine diseases by analyzing abnormal behavior patterns through wearable sensors [9,10,11]. By attaching an activity sensor to a dog in a manner that does not hinder their daily activities, monitoring becomes more accessible, facilitating the detection of abnormal patterns. Activity sensors, traditionally used to monitor human health and broadly implemented in areas such as rehabilitation, healthcare, and health indicators [12], are now being adapted for canine health. Previous research in humans has demonstrated that dietary therapy and tailored health management, aided by activity sensors, significantly enhance health and quality of life through continuous monitoring and wellbeing scores [13]. While extensive studies have assessed human health statuses [14], the development of healthcare programs for dogs faces challenges due to variables such as breed, age, and weight, which are difficult to quantify with simple data patterns.

This study introduces an advanced activity sensor, incorporated into a multifaceted algorithm, to measure activity levels based on the specific behaviors of companion dogs. The objective is to devise a method for deducing canine health statuses. We collected data on representative behavioral changes correlating with each dog’s health status. Their health status was inferred using a health score, employing a data-based algorithm to identify abnormal patterns. This process involves learning basic data over a period, detecting activity-specific data, and estimating activities within a defined range. Dog behavior varies with breed, size, weight, and age, even in identical environments. To address this, data were analyzed and controlled in a multifaceted manner. Following this, a Health Score was developed to assess the dogs’ health status based on collected data. It is crucial to recognize that the pattern of disease-related abnormal behavior in dogs can vary not only by breed and weight but also according to the surrounding environment. Hence, accurate data extraction and baseline establishment require setting a standard over a specific period by correlating observed image data with disease data. Establishing these quantitative standards enables various application-based approaches. An app was developed to enhance the visual understanding and accessibility for individuals without veterinary knowledge, aiming to facilitate health monitoring and commercialization in the future.

This research endeavored to conduct a multifaceted data analysis, extending beyond mere data utilization to modifying certain predictable data. To achieve this, we employed the fuzzy associative memory technique, utilizing over 600 learning data instances encompassing four behavioral patterns, stratified by dog and weight, from animal hospitals. This approach aimed to predict abnormal behavior, which is assessed based on the individual criterion of the frequency of occurrence and can be characterized as either significant or negligible. This study posited that the presence or absence of a disease could be predicted by correlating patterns of abnormal behavior, exceeding a quantitatively established threshold of learned behavioral data, with potential diseases. These correlations were quantified to formulate the Health Score. The findings of this research are anticipated to lay the groundwork for determining the health status of dogs based on abnormal behavior patterns.

## 2. Materials and Methods

### 2.1. Dog Sample

The duration of the study was nine months, spanning from May 2022 to February 2023. The selection of canine participants involved the use of dog parks, dog cafes, and veterinarian recommendations. Criteria for inclusion encompassed dogs exhibiting normal daily life levels, those without any surgical history in the preceding three months, and dogs under the age of 10, as determined by dental and joint assessments by veterinarians. Ultimately, the study was conducted on a total of 30 dogs. To ensure a precise understanding of existing diseases and health conditions prior to the commencement of the experiment, all participating dogs underwent a health checkup at a veterinary hospital to ascertain any pre-existing conditions. For analytical purposes, dogs were categorized based on weight into the small, medium, and large groups, and further classified by age. The distribution of these categories by weight and age is detailed in Table 1.

### 2.2. Data Collection and Environment

**Figure 1:**
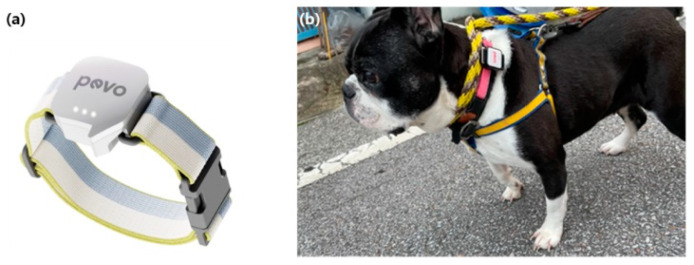
Sensor design and placement: (**a**) acceleration sensor’s appearance, (**b**) sensor placement on dog’s neck.

In this study, activity sensors were attached to dogs to gather data. The employed sensor is a validated, reliable device, detailed in Figure 1. It measures 33 mm by 38 mm, has a thickness of 18.3 mm, and weighs 15 g. Its lightweight, compact design ensures minimal interference with the dogs’ daily activities and prevents discomfort. The sensor was affixed to the dogs’ necks using a collar, facilitating easy movement tracking. It features Bluetooth 5.0 connectivity, IP67 waterproof certification, a 2000 degrees per second acceleration sensor, a gyro-sensor resolution of 50 Hz, and can measure up to 2G.

**Figure 2:**
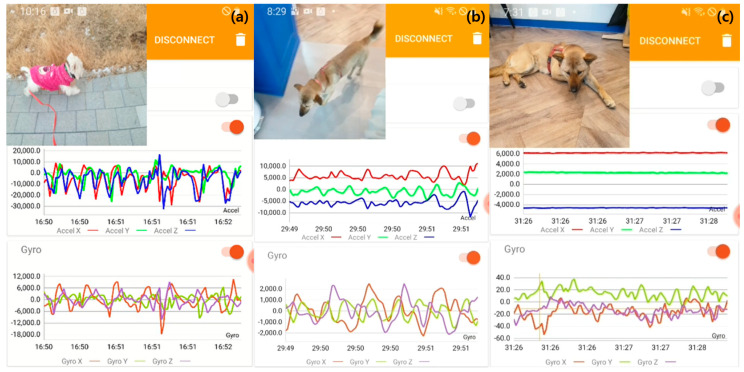
Interface of the sensor data application: (**a**) display of running activity data, (**b**) display of walking activity data, and (**c**) display of lying activity data.

To ascertain the accuracy of the data captured by the sensor, a concurrent recording approach employing a smartphone camera was utilized. The behaviors monitored for validation were categorized into dynamic and static types. Dynamic behaviors in companion dogs include activities such as running, walking, jumping, eating, and swallowing. In contrast, static behaviors are those where the dog’s position remains constant, such as sitting, lying, and sleeping [15]. Data collection was time-zoned based on the dogs’ activity levels. The activity measurements derived from smartphone imagery and the acceleration and gyro-sensor data underwent a filtration process, as depicted in Figure 2. This figure illustrates the application interface used for sensor data filtering. Filtering criteria were established by correlating the sensor data values with the actual images, enhancing data accuracy and completeness.

The behavioral data integral to this study comprised scratching, licking, swallowing (both food and water), and sleeping. Initially, three months of baseline daily behavior data were collected from each dog. These four behavioral data types, gathered from over 600 instances, were processed through the algorithm for training and utilized in a comparative analysis with multifaceted data matching. The three-month data collection period enabled the learning algorithm to classify these behaviors as indicative of normal and healthy patterns. Any dogs exhibiting abnormalities during this period were excluded from the experiment following veterinarian consultation. The learning algorithm utilized an associative memory algorithm for its learning mode. This algorithm filters similar patterns and employs associative techniques to match identical data after confirming the behavioral analysis of the dogs [16]. The behavioral sensor on the dogs identified four key behaviors, which were established as reference points for each dog breed through veterinary consultation, as outlined in Table 2. For the behaviors of scratching and licking, we employed a four-tiered classification system: occasional, average, frequent, and severe. Consequently, minimal scratching and licking detected by the sensor suggest a healthy state free from disease, whereas excessive occurrences of these behaviors may indicate skin diseases [17]. In addition to scratching and licking, this study also calculated the dogs’ Health Score based on swallowing and sleeping behaviors. Table 3 presents the standard data metrics for these activities. The level of swallowing activity was quantified by the number of swallows per hour. A count lower than the average level, as measured by the sensor, may suggest digestive diseases or esophageal disorders, while a higher count could indicate excessive water intake, potentially symptomatic of chronic kidney disease [18].

Therefore, the pattern of swallowing can serve as an indicator of abnormalities. Sleep data were assessed based on the average daily duration of sleep for a companion dog, typically ranging from 12 to 16 h [19]. Consequently, reduced sleep can be indicative of insomnia or other health issues [20]. Standards for each behavior were established after considering the risk factors and normative benchmarks. Baseline health data for the dogs were acquired from veterinarians, and this information was processed to compute the Health Score through AI-driven data analysis. The anomaly detection algorithm was developed using a neural network approach combined with fuzzy associative memory. This method involved correlating symptomatology with diseases, training the system using 600 instances of data from dogs with skin and digestive diseases of the same breeds from existing veterinary clinics, and then comparing this with the gathered data, starting with data matching. However, due to inconsistencies in the basic data regarding the breeds, ages, and weights of the experimental dogs, we adapted the fuzzy associative memory technique to be breed-specific.

**Figure 3:**
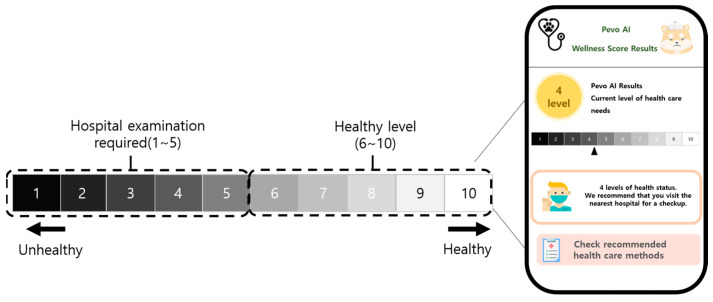
Visualization of the health score in the application.

The Health Score is a quantitative metric that was developed to facilitate the visualization of initial activity analysis results. This score reflects the health status of the dog and provides a straightforward indication of potential changes based on future data [21]. By integrating the dog’s baseline data with sensor-acquired data, the Health Score is computed on a scale from 1 to 10. As illustrated in Figure 3, a score of 1 signifies a low health level and an elevated risk of disease, while higher scores denote better health. AI-predicted Health Scores ranging from 1 to 5 suggest existing health conditions that require medical attention. Scores between 6 and 10 are indicative of a generally healthy state. The validity of these assessments was corroborated by three veterinarians, who compared the AI-generated scores with the AI’s learning outcomes [22].

**Figure 4:**
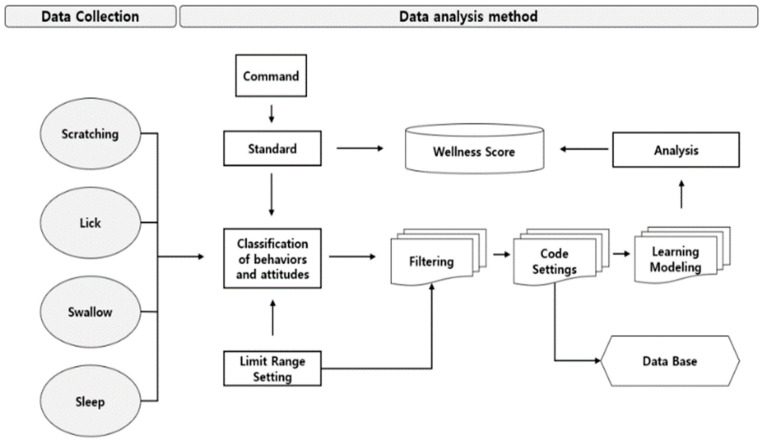
Procedure for establishing the Health Score criteria.

Figure 4 demonstrates the process of calculating the Health Score, which involves filtering the analytics data according to the established behavioral pattern criteria. Equation (1) presents the calculation formula, incorporating the result value’s weight (*W*), reference data, and the associative memory learning model [23]. In this equation, *i* denotes the data from the activity sensor, *W* denotes the data weight for each behavior, and *j* represents the data from the Health Score calculation, adjusted based on their variation from existing data. By defining correlations between reference data sets, potential data conflicts were preemptively addressed. For data repeating over time, the homogeneous associative memory technique was employed to integrate the learned data into the Health Score. Additionally, repetitive data, over time, were utilized as learning data proportional to the Health Score in a healthy state, facilitated by the application of homogeneous memory techniques. Hence, the final result data, reflecting behavioral changes, can be continually updated through the repeated learning of activity and change data, as delineated in Equation (2).

(1) $$𝒾=\sum_{𝒾=0}^{n-1}(w_{a}+w_{b}+w_{c}+w_{d}),\;𝒿=\sum_{𝒿=0}^{n-1}{\left({𝒳}_{𝒾}+{𝒳}_{𝒾𝒿}\right)}^{2}=W_{𝒿},$$

(2) $$W_{(a+b+c+d)𝒾𝒿}=\sum_{𝒾=1}^{n}\left({𝓍}_{𝒾}\right)\sum_{𝒿=1}^{n-1}\left({𝓍}_{𝒾}\right).$$

This study calculated a Health Score based on the dogs’ activity levels. To validate the resultant Health Scores, we compared them with the health status assessments made by the three veterinarians who conducted the initial health examinations. The AI-generated Health Score is a form of categorical (ordered) data, divided into two primary categories: dogs requiring medical attention and those in good health. The veterinarians were also asked to classify the dogs into these categories. The congruence between the classifications made by the veterinarians and the AI was analyzed using Equation (3) [24]. While maintaining canine health is crucial, it is equally important to analyze both dynamic and static data from behavioral sensors to identify unhealthy conditions and predict potential diseases, aiming for 100% sensitivity in disease detection [25]. Accordingly, the three veterinarians evaluated each dog’s health status and compared the level of agreement, particularly in cases of abnormal health conditions.

(3) $$percent\;agreement\left(\%\right)=100\times \frac{a+d}{a+b+c+d}.$$

Veterinarian and AI app are positive: a

Opinions of veterinarians and AI apps are different; positive, negative: b, c

Veterinarian and AI app are negative: d

## 3. Results

Table 4 details the general characteristics of the companion dogs involved in this study. The average age range of the dogs was 3–6 years. Among the 30 dogs, 16 were male and 14 were female. The average weight varied significantly, ranging from 3 kg to 30 kg, with Jindo dogs and poodles being the heaviest. The remaining dogs were of mixed breeds. Accurately determining the breed based solely on the assertions of dog owners and veterinarians can be challenging.

The dogs were equipped with sensors on their collars, and their daily activities over six months were analyzed. Four types of data were evaluated according to dog breed: scratching, licking, swallowing, and sleeping. As indicated in Table 5, the average scratching frequency for retrievers, Jindo, and Shih Tzu breeds was 122, 130, and 144 times, respectively. Their scratching and licking patterns exceeded the standard by 18, 22, and 16 times, respectively. Poodles exhibited a swallowing frequency of 74 times, which was higher than the average for other breeds. Beagles and mixed breeds showed swallowing frequencies of 26 and 23 times, respectively. All breeds, except retrievers, Shih Tzus, and Jindos, averaged more than 12 h of sleep, aligning with the typical range.

The comparison between the Health Scores generated by the algorithm and the diagnoses made by veterinarians is presented in Table 6. For certain breeds such as retrievers, pit bulls, and Shih Tzus, the algorithm assigned health scores ranging from 3 to 4, indicating a health status that necessitates veterinary examination. These scores aligned with the veterinarians’ assessments, which also indicated health conditions requiring specific diagnostic attention. Additionally, the AI-derived health scores corresponded with the veterinarians’ diagnoses for Siberian huskies, poodles, Maltese, and other breeds. However, a discrepancy was noted in the case of the beagle. While the AI algorithm suggested a health score of 4, recommending a health test, the veterinarian assessed the beagle as healthy. Despite this variance, the overall agreement between the AI-evaluated results and the veterinarians’ analyses was 87.5%. This level of concordance generally indicates that the Health Scores are in line with the health evaluations conducted by veterinarians. Consequently, this outcome substantiates the efficacy of the quantitative Health Score system in reflecting the behavioral patterns of companion dogs. This study’s findings underscore the potential of this AI-driven approach in aiding the assessment of canine health and supporting the early detection of health issues.

## 4. Discussion

The advent of ICT has facilitated various methods for remotely evaluating a dog’s health status. Recent research has indicated the feasibility of predicting or assessing a dog’s health condition in advance [26]. Nonetheless, it is critical to evaluate the reliability of these results and the effectiveness of the measurement methodologies used. To address the limitations inherent in traditional questionnaires and subjective assessments, this study introduced a novel approach, comprising sensor-based data collection, AI-based analysis of similar behavior patterns, and a quantitative expression method, namely the Health Score. The validity of the results was ascertained through comparison with diagnoses made by veterinarians. The Health Score emerged as an effective tool for articulating the health status of companion dogs [27]. For dog owners lacking veterinary expertise, visually assessing a dog’s health can be challenging; therefore, the developed method holds significance in evaluating a dog’s condition based on daily activities. Customized data analysis through sensor-based and AI methods, focused on the behavioral observation of companion dogs, provides a robust means of inferring a dog’s health status from repetitive behaviors like scratching, licking, and swallowing. However, the connection between current and optimal health states is limited, necessitating the analysis of historical data.

This study is not without its limitations. First, the predictions were solely based on data analysis, lacking comprehensive veterinary data such as health conditions, coat quality, and age, which are typically observable with the naked eye. Second, the International Canine Federation recognizes over 300 breeds of companion dogs [28], yet this study encompassed only eight breeds. Third, the research was conducted over a nine-month period with a sample size of only 30 companion dogs. To overcome these challenges, future research should aim to include a more diverse array of breeds by recruiting a larger cohort of subjects. Additionally, the development of advanced sensors and the exploration of fuzzy hierarchical techniques are recommended to enhance the veterinary data and improve prediction accuracy.

This study’s application of a learning model and artificial intelligence program hinges on the quantitative standard of frequency, predicated on the notion that dogs exhibit a limited range of behaviors, with similar patterns potentially indicative of health issues. Associative memory techniques utilize this similarity in behavior patterns, suggesting that identical behaviors could have analogous meanings. Thus, in canines, disease patterns might be discernable through associations drawn from repetitive actions.

The app developed in this research displays the health score in two formats: as a range and as a numerical value. However, this study primarily presented the score as a range. This approach was chosen to reflect the current health status in light of various health factors that cannot be quantified precisely. It is designed to prevent misinterpretation of the health status due to fluctuating behavior patterns, allowing for more realistic judgment by the dog owner.

The AI analysis algorithm demonstrated a high level of agreement in predicting the health status of the study subjects. Furthermore, the use of small, lightweight sensors ensured the successful observation of companion animal behaviors without causing discomfort or posing biological risks to the dogs. These findings are anticipated to be valuable for future pet-related research employing similar sensors, particularly in modern societies with growing pet populations. Such studies could enhance communication between dog owners and their pets.

Continuous, data-based health monitoring is crucial. The Health Score reflects the current health status, and if no abnormal behavior is detected over a day, the results are displayed and then archived as historical data. It is important to note that the Health Score in this study does not diagnose or predict diseases; instead, it monitors the current health status. The score is presented not as a definitive quantitative value but as a range, indicative of the general health status checked, thereby providing a more nuanced understanding of the dog’s wellbeing.

When a sensor attached to a dog exhibits no response or demonstrates a data rhythm that deviates from established patterns, the system is designed to filter out abnormal data preceding and succeeding the initial starting point. This process ensures that anomalies in sensor readings, which could indicate either a malfunction or a significant change in the dog’s behavior, are identified and addressed. Moreover, the system isolates and eliminates changes in behavior through iterative data analysis. This method of parsing out irregularities not only maintains the integrity of the data collected but also enhances the accuracy of behavioral assessments. The advancement in sensor technology, particularly in recognizing behavior patterns, opens new avenues for future developments in this field. The use of advanced platforms in sensor technology can lead to more sophisticated, sensitive, and reliable methods of monitoring and interpreting animal behavior. Such progress holds significant potential for improving the quality of pet healthcare, enabling the more precise and timely detection of health issues in companion animals. As sensor technology continues to evolve, it may offer increasingly nuanced insights into animal behaviors and health statuses, thus aiding pet owners and veterinarians in ensuring the wellbeing of companion animals.

## 5. Conclusions

In this study, a Health Score system was developed to provide a quantitative assessment of the health status of companion dogs. This system utilized basic data, including the breed, age, and weight of the dogs, along with both dynamic and static daily data, gathered through activity sensors. The Health Score, derived from an AI algorithm, was formulated by analyzing and learning from data representing normal and healthy canine behaviors. The Health Scores, reflecting the AI-estimated quantitative health status of the companion dogs, demonstrated a high degree of accuracy, aligning with the diagnoses made by veterinarians at a rate of 87.5% concordance. This outcome underscores the efficacy of the Health Score as a reliable tool for health assessment. Consequently, a methodology for measuring and calculating Health Scores based on the daily life data of companion dogs was proposed. This innovative approach is particularly beneficial for individuals who lack the ability or find it challenging to assess their dog’s health independently. Using technology and AI, the Health Score system offers a practical, data-driven solution for pet owners to monitor and understand the health and wellbeing of their canine companions. It represents a significant step forward in pet healthcare, enabling more the proactive and informed management of companion animal health.

## Figures and Tables

**Table 1:** Classifications for participating dogs.

**First Classification**	
**Size**	**Weight**
Large dog	Over 15 kg
Medium dog	7–15 kg
Small dog	Less than 7 kg
**Second Classification**	
**Life Cycle**	**Age**
Adulthood	8–10 years old
Adolescence	3–7 years old
Babyhood	0–2 years old

**Table 2:** Criteria for analyzing sensor measurements of scratching and licking activities.

Amount of Scratching Activity		Amount of Licking Activity	
Standard	Times per Hour	Standard	Times per Hour
average	0–52	average	0–7
sometimes	53–119	sometimes	8–19
often	120–299	often	20–43
serious	over 300	serious	over 44

**Table 3:** Criteria for analyzing sensor measurements of swallowing and sleeping activities.

Amount of Swallowing Activity		Sleep Data	
Standard	Times per Hour	Standard	Time to Sleep
below average	0–30	deep sleep	more than 12 h
average	31–59	a little bit of trouble	−20% of a good night’s sleep
above average	over 60	strikingly little	−70% of sound sleep

**Table 4:** Analytical results of companion dogs participating in the study.

Assortment	Dog Breed (Number of Dog)	Average Age	Gender (Male/Female)	Average Weight (kg)	Classification (Size)
participating dog breeds	Retriever (3)	4.8	1/2	26.2	large
	Siberian Husky (3)	5	2/1	26.6	large
	Jindo dog (5)	4.2	2/3	17.8	large
	Poodle (5)	5.3	3/2	9.3	medium
	Beagle (2)	3.5	2/0	7.8	medium
	Maltese (4)	4	2/2	4.6	small
	Shih Tzu (4)	5	3/1	5.7	small
	Other (4)	4.5	2/2	9.8	medium

**Table 5:** Detection of dog behavior patterns by sensors.

Measured Behavioral Items (Average)	Average Count of Sensor Detection by Breed (Number of Dogs)							
	Retriever (3)	Siberian Husky (3)	Jindo Dog (5)	Poodle (5)	Beagle (2)	Maltese (4)	Shih Tzu (4)	Other (4)
number of scratches	122	42	130	53	37	40	144	46
number of licks	18	8	22	5	9	7	6	3
number of swallows	42	58	34	74	26	61	50	23
sleep time	9.7	12.5	8.7	13	13.2	14.2	10.2	12.4

**Table 6:** Comparative analysis of dog health scores: algorithmic calculation vs. veterinarian diagnosis.

	Participating Dog Breeds (Number of Dogs)	Concordance				Health Level			Total
			AI		Total	A1		Veterinarian	
		Veterinarian	Positive	Negative		Health Score	State		
Health by breed level	Retriever (3)	Positive	3	0	3	4	examination required	examination required	correspondence
		Negative	0	0	0				
	Siberian husky (3)	Positive	3	0	3	9	healthy	healthy	correspondence
		Negative	0	0	0				
	Jindo dog (5)	Positive	5	0	5	3	examination required	examination required	correspondence
		Negative	0	0	0				
	Poodle (5)	Positive	5	0	5	8	healthy	healthy	correspondence
		Negative	0	0	0				
	Beagle (2)	Positive	2	0	2	4	examination required	healthy	inconsistency
		Negative	0	2	2				
	Maltese (4)	Positive	4	0	4	8	healthy	healthy	correspondence
		Negative	0	0	0				
	Shih Tzu (4)	Positive	4	0	4	4	examination required	examination required	correspondence
		Negative	0	0	0				
	Others (4)	Positive	4	0	4	9	healthy	healthy	correspondence
		Negative	4	0	0				
Total									87.5%

## Data Availability

All data generated or analyzed during this study are included in this published article.
